# Low-Malignant Schwannoma of the Cervix in Pregnancy: A Case Report and Literature Systematic Review

**DOI:** 10.1155/2021/6806960

**Published:** 2021-12-09

**Authors:** Giada M. ALMIRANTE, Francesco CANTATORE, Gianluca TACCAGNI, Massimo CANDIANI, Massimo ORIGONI

**Affiliations:** ^1^Department of Obstetrics & Gynaecology, Vita Salute San Raffaele University School of Medicine and IRCCS San Raffaele Scientific Institute, Milano, Italy; ^2^Department of Pathology, Vita Salute San Raffaele University School of Medicine and IRCCS San Raffaele Scientific Institute, Milano, Italy

## Abstract

Primary cervical melanocytic schwannomas, arising from the sympathetic chain, are very rare pigmented neural sheath tumors that, both grossly and clinically, are often misdiagnosed with other more frequent lesions of the uterine cervix. Literature review accounts for seventeen published cases of schwannomas of the cervix, ten of which are malignant and seven benign. Pathological examination with immunostaining techniques is essential for the correct diagnosis of these tumors. We report a case of primary cervical schwannoma in a 32-year-old female who was referred to our department at 13 weeks of gestation with a diagnosis of malignant melanoma of the cervix. Pathological review detailed a neoplasm with a myxoid spindle cell component and a minority of small epithelioid cells, with a low-malignant potential proliferation index: morphological and immunocytochemical findings were compatible with the diagnosis of nerve sheath tumor, type schwannoma. The patient underwent a vaginal trachelectomy and a prophylactic Shirodkar's cerclage. Pregnancy was carried on without any negative event. The patient delivered by a caesarean section a healthy female newborn. Placental histology was negative. After 6 years from the first diagnosis, the patient is healthy and disease free.

## 1. Introduction

Schwannoma, otherwise known as neurinoma or neurilemmoma, is a particular type of neurogenic tumor. It is described as an encapsulating tumor, originating from the Schwann cell of the peripheral nervous system and first described by Verocay in 1908 [1]. It is slow growing and can occur anywhere along any somatic or sympathetic nerve in the body except the olfactory and optic nerves, which lack Schwann cell sheaths and which are part of the central nervous system. About 25–45% of all schwannomas are found in the head and neck region; they are also reported to occur at the upper and lower extremities, posterior mediastinum, and retroperitoneum. Primary cervical schwannomas, arising from the sympathetic chain [2], are very rare pigmented neural sheath tumors that, both grossly and clinically, are often misdiagnosed with other more commonly detected lesions of the uterine cervix. Pathological examination with comprehensive immunostaining techniques is essential for the diagnosis of these tumors. Because there are no specific clinical or radiological signs for pelvic schwannomas and they resemble a number of pelvic lesions, misdiagnosis is likely to occur. Being a rare disease entity with only a few available published reports, the characteristic features, clinical presentations, treatment, and outcomes are not clearly understood. This article is aimed at presenting a report of a significant clinical case and at increasing the available literature on schwannomas arising from the uterine cervix.

## 2. Case Report

**Figure 1:**
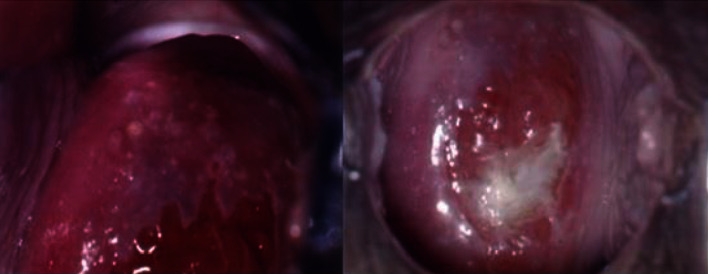
Colposcopy: normal cervix.

**Figure 2:**
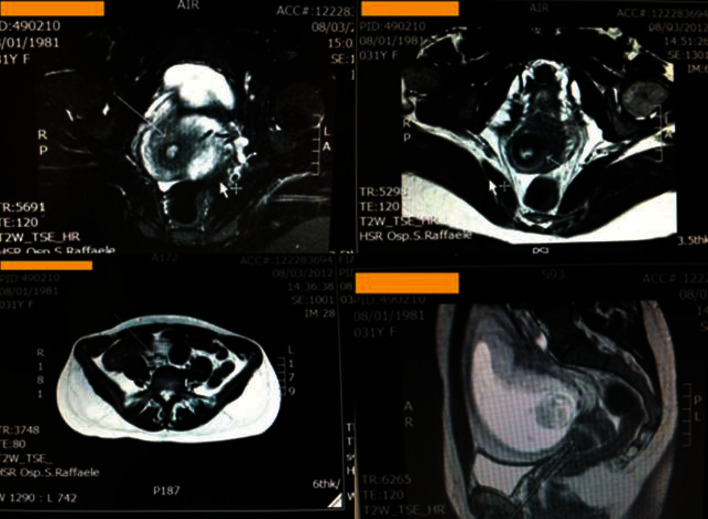
MRI mild-signal hyperintensity in the posterior-left and front cervical side (arrows); no aspects of infiltration of the parametrium.

**Figure 3:**
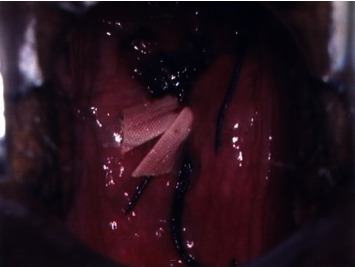
The cervix after vaginal trachelectomy and Shirodkar's cerclage.

**Figure 4:**
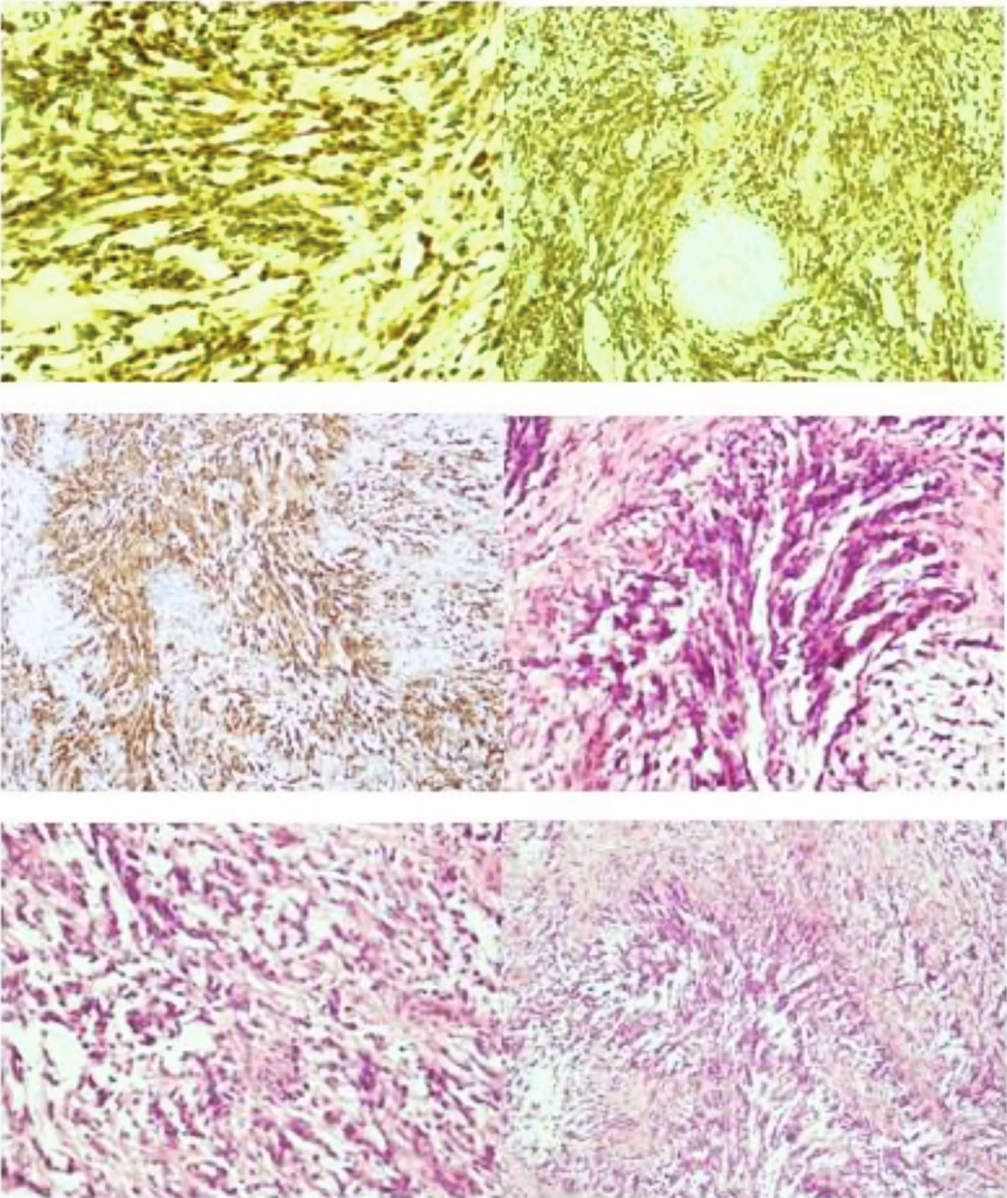
Immunohistochemical positive staining for protein S100 and GFAP.

We report a case of primary cervical schwannoma in a 32-year-old woman who was referred to our department with a diagnosis of malignant melanoma of the cervix in pregnancy. Before referral, she underwent a colposcopy with targeted biopsy because of a white, thick, and hardened round lesion of the left upper margin of the cervix detected by vaginal manual exploration. Biopsy pathological exam revealed a minimal fragment of mesenchymal proliferation with areas of myxoid degeneration together with regular round core elements, no apparent mitosis, no necrosis, and further excision recommendation for actual analysis. A pathological slide review was performed, and identification of a neoplastic lesion with spindle and epithelioid cells obtained was performed. In the meantime, the patient became pregnant. The immunophenotypic characterization was consistent with a diagnosis of malignant mucous melanoma. At 8^+5^ weeks of gestation, the patient was submitted to a loop electrosurgical excision procedure (LEEP) of the cervix which revealed an ulcerated tumor with spindle and epithelioid cells. Immunocytochemistry stained positive for vimentin and S100 protein and negative for desmin, anti-muscle actin antibody (HHF35), cytokeratins, Estrogen Receptor (ER), Progesterone Receptor (PR), Epithelial Membrane Antigen (EMA), synaptophysin, chromogranin, and smooth muscle actin; a low proliferative activity was detailed; surgical conization margins were positive. An additional pathological review was carried out and obtained at 11^+2^ gestational weeks: this revealed a morphological and immunocytochemical framework consistent with infiltrating melanoma of the cervix. Immune reactivity with antiprotein S100 and Melanoma Marker Antibody (anti-HMB45) was positive, while that with antityrosinase1 and anti-MITF was negative. With all this information, the patient, now at 13^+6^ weeks of her pregnancy, was referred to our department. On admission, she underwent chest X-ray, colposcopy, complete abdomen Magnetic Resonance Imaging (MRI), psychological counselling, gynaecological oncological ultrasound, obstetric ultrasound, and immunopathological review of the conization specimen. Colposcopy showed a normal cervix (Figure 1), with areas consistent with the recent LEEP procedure; the Squamous-Columnar Junction (SCJ) was fully visible, and no atypical area was detected. Application of 5% acetic acid did not reveal any positive aceto-whitening area; it only detected three yellowish-white nodular round formations, non-aceto-reactive, consistent with a nabothian cysts. A pelvic ultrasound scan showed, at the front cervical lip, in the anterolateral left side, a hypoechoic area with increased vascularization (color score 4) of 10 × 8 × 25 mm. That reached the cervical canal and the left margin. The left parametrium appeared slightly thickened, measuring 30 × 26 mm. An obstetrical ultrasound scan was negative with normal fetal nuchal translucency (NT). The patient decided to perform chorionic villus sampling (CVS) that showed a female 46XX normal karyotype. The abdomen-pelvis MRI scan (Figure 2) did not show any morphological changes of the abdominal organs. The cervix did not present patterns suggestive for expansive lesions: a mild hyperintense signal in the posterior-left and front side, which reached the cervical outer border, was recorded, but no aspects of infiltration of the parametrium were observed. The left vaginal fornix was minimally thickened but without any abnormal signal, probably due to the lateral deviation of the cervix. No pelvic and/or lumbar-aortic adenopathy was recorded; both ovaries were normal. On the basis of the complete negativity of all the diagnostic procedures performed, we decided to resubmit the previous pathological specimens to further review. Our pathologist described positive staining for protein S100 and weak Glial Fibrillary Acidic Protein (GFAP) positivity; staining for tyrosinase, melanA, HMB45, pool of cytokeratins, EMA, desmin, and neurofilament was negative. Microscopically, the lesion was characterized by proliferation of irregular fascicles or strands, sometimes intersecting, of cells in a fibro-myxoid or sclerotic stroma; cells were spindle-shaped, with elongated nuclei, and irregular in shape or twisted, with a moderate amount of the cytoplasm at the cellular poles. A small amount of epithelioid cells with a low-malignant potential proliferation index (immunocytochemistry MIB-1 proliferation index < 10% and mitotic index < 2 mitosis for 10 High-Power Fields (HPF) at 400x magnification) was described; no lymphovascular space invasion (LVSI) was detected; morphological and immunocytochemical findings were compatible with a nerve sheath tumor, type schwannoma (Figure 3). According to this last pathological diagnosis and due to the lack of gross clinical information of the primary lesion excised, a pathological staging was not feasible. At 14^+4^ weeks, after a detailed counselling, including the hysterectomy option, the patient accepted to be submitted to a vaginal trachelectomy and prophylactic cervical cerclage according to the Shirodkar technique (Figure 4). The pathological report of the trachelectomy specimen was negative for any previously described features of schwannoma, and surgical margins were negative. The patient was then monthly followed at the obstetrical outpatient unit, and the pregnancy proceeded without any issues. At 38^+3^ weeks, she surgically delivered by a C-section a female newborn of 3300 g of weight; the Apgar score was 10-10, and the newborn blood tests were all normal. Placental pathology was normal. The patient was discharged from hospital on day 4 after delivery and was enrolled in a follow-up program according to our protocols for cervical diseases: cytology, colposcopy, and pelvic imaging (gynaecological ultrasound and abdomen-pelvis CT scan). After 6 years from the first diagnosis, the patient is healthy and her well-being is good without any evidence of cervical disease.

## 3. Discussion

As far as we know, this is the first described case in literature of a cervical schwannoma diagnosed and treated in pregnancy. Melanocytic schwannoma is a rare nerve sheath tumor that occurs in a wide variety of locations. Most commonly, it occurs in middle aged subjects, without a clear gender predilection [3]. These tumors are usually detected as a solitary, painless, and slow-growing mass of variable size [4]. They usually involve superficial soft tissues of the extremities, relatively most often in the head and neck region and the distal parts of the extremities. Uncommonly, large tumors are found in the posterior mediastinum or the retroperitoneum, as described in several reports [2, 4–7]. When located in the pelvic cavity, schwannomas are usually misdiagnosed as gynecologic masses. Typical schwannomas can also primarily involve visceral sites such as the gastrointestinal tract, the kidneys, and the breasts; however, their diagnosis in the uterine cervix is extremely rare [2, 3]. They are usually asymptomatic until their massive growth compresses adjacent organs. Multiple similar lesions may be seen in association with von-Recklinghausen's neurofibromatosis. Schwannomas are mostly benign and less than 1% progresses towards malignancy transformation, known in these cases as malignant peripheral nerve sheath tumors (MPNSTs) [2]. Primary malignant schwannomas are a type of neural sheath tumors, representing 10% of soft tissue sarcomas. They are remarkable for their aggressive growth. The median age of patients at onset is 36 years, with a typical high prevalence between the ages from 20 to 50 years (8). Among all schwannomas, the pelvic occurrence represents 1–3% of all cases [5]. The melanin production by Schwann cells is explained by the common origin of melanocytes and Schwann cells from neural crest cells. The clinical behaviour of this neoplasm is variable. Conditions that should be considered in the differential diagnosis are neurofibromas, leiomyomas, angiomyofibroblastomas, and melanomas [3]. The histological cell types of malignant schwannomas include glandular, melanocytic, malignant triton, and epithelioid types. The schwannoma is characterized by alternating areas of hyper- (Antoni A) and hypo- (Antoni B) cellularity. Clusters of parallel-arranged spindle cells are contained in Antoni A areas, forming palisades, commonly known as Verocay's bodies. Cells loosely arranged in a myxoid background are present in the Antoni B areas [8]. The absence of staining for CD34 and neurofilament protein (NFP), together with the strong positivity for S100 protein, supports the diagnosis of schwannoma as opposed to neurofibroma. Moreover, the presence of thickened-wall hyalinized blood vessels favours the diagnosis of schwannoma. Positivity for the S100 protein and negativity for desmin and actin argue against leiomyoma and angiomyofibroblastoma. Finally, a negative pan-melanoma staining essentially excludes melanoma [9–13]. Preoperative neurologic findings, anatomic location, electron microscopy, and immunohistochemistry findings help to establish the diagnosis between this neoplasm and the more common malignant melanoma, with electron microscopy having a role in distinguishing between benign and malignant lesions. Literature review accounts for seventeen published cases of schwannomas of the cervix (Table 1), ten of which were malignant. Malignancy in schwannomas or MPNST typically presents with abnormal vaginal bleeding [14]. As far as it regards our reported case, the rationale behind the decision to perform a simple trachelectomy, rather than a radical procedure, was guided by several coexistent factors: first of all, the lack of any evidence of malignancy either preoperatively or from the pathological reviews of the first-line conservative excisions, and secondly, the ongoing pregnancy at the time of the patient's referral to our department played a significant role in our, together with the patient's willingness, decision-making process. Commonly, surgical excision is recommended in cases of benign schwannomas, which rarely recur if completely excised [4]. The surgical approach to a mesenchymal neoplasm of the cervix, in fact, especially in young patients, actually requires a careful planning; conservative conization may be considered with the double meaning of a diagnostic procedure and the possible therapeutic role in cases of benign or noninvasive lesions. Hysterectomy, as described by Di Giovannantonio et al. [15], it may be considered in women in nonreproductive ages or in cases of infiltration of surgical resection margins in nonpregnant women. All reported cases of cervical malignant schwannoma have been treated with total abdominal hysterectomy. Our case is the first description of a low-malignant cervical schwannoma treated by local excision. Patients diagnosed with malignant schwannoma have a guarded prognosis. Metastatic disease is present in 16% of patients at the time of diagnosis, with the lungs being the most common site of metastasis. Malignant schwannoma has a high rate of local recurrence and a predilection to occur in sites of prior radiation. The 5-year survival rate has been reported as 47–65%, with an average survival of 24–48 months. Poor prognosis is associated with concomitant NF-1, tumor size greater than 5 cm, and younger age 5of patients at diagnosis. Better clinical prognosis is associated with surgical resectability, tumor size less than 5 cm at presentation, low histological grade, and patients' age higher than 30 years old at diagnosis [9]. A preoperative diagnosis of this tumor may be challenging because of its rarity as well as the various differential diagnoses, which must be considered by both gynecologists and pathologists involved. Several studies have shown that FDG PET or PET/CT represents a sensitive technique in the detection of malignant schwannomas in patients with neurofibromatosis type 1. In addition, FDG PET can be used to guide biopsy, in order to plan the appropriate therapy, for the staging of the disease, restaging, and posttherapy follow-up of malignant schwannomas [14].

## 4. Conclusion

Although the uterine cervix is a rare site for schwannomas, it should be considered in the differential diagnosis of any unclear cervical mass. Because of their rarity, the diagnosis of these tumors remains a challenge for both clinicians and pathologists. Our reported case underlines the pivotal importance of the pathological differential diagnosis among several conditions sharing similar aspects with schwannomas and melanomas. Currently, complete surgical resection is the mainstay of treatment, and the goal of radicality should always guide the decision-making process. Nevertheless, in specific circumstances, especially when a comprehensive clinical and pathological workout does not confirm any evidence of malignancy or high-malignant potential in young patients, a conservative choice may, in our opinion, be safely considered. As our experience represents a single and isolated case, we do not have any supporting element to recommend such a choice and we advocate for the need of more similar reports to be published and shared within the scientific community.

## Figures and Tables

**Table 1:** Published cases of cervical schwannomas.

Author	Age	Presentation	Pathology	Treatment	Outcome
Cid [16]	NA	NA	Benign schwannosis	NA	NA
Gwavava [11]	38	Nodule	Benign schwannoma	DE	NA
Terzakis [12]	47	Nodule	Melanocytic schwannoma	TAH	NED 3 years FU
LeMaire [17]	47	Nodule	Benign schwannoma	LEEP	NED 6 months FU
Tahmasbi [3]	48	Uterine mass	Benign schwannoma	TAH BSO	NA
Sloan [18]	47	AVB	Malignant schwannoma	TAH BSO	NED 1 year FU
Junge [19]	41	AVB	Malignant schwannoma	TAH PLNS	NA
Keel [20]	25/65/73	1 & 2: AVB 3: polyp	Malignant schwannoma	TAH	1 & 2: NA 3: DP
Lallas [13]	51	AVB	Malignant schwannoma	TAH BSO	NED 1 year FU
Bernstein [9]	65	AVB	Malignant schwannoma	RH PPLND	NA
Di Giovannantonio [15]	27	AVB	Malignant schwannoma	TAH	NED 3 years FU
Kim [21]	50	Polyp	Malignant schwannoma	RH BSO PLND ACT	DP LFU
Dong [14]	45	AVB	Malignant schwannoma	LRH BSO PLND	NA
Dey [4]	37	Polyp, AVB	Benign schwannoma	D&C	NED 6 months FU
Present case	32	Nodule	Low-malignant schwannoma	LEEP-VT	NED 6 years FU

ACT: adjuvant chemotherapy; AVB: abnormal vaginal bleeding; BSO: bilateral salpingo-oophorectomy; DE: diathermy excision; D&C: dilation and curettage; DP: disease progression; FU: follow-up; LEEP: loop electrosurgical excision procedure; LFU: lost at follow-up; NA: not available; NED: no evidence of disease; PLND: pelvic lymph node dissection; PLNS: pelvic lymph node sampling; PPLND: pelvic and periaortic lymph node dissection; RH: radical hysterectomy; TAH: total abdominal hysterectomy; VT: vaginal trachelectomy.
